# Alteration of the Gut Microbiome in Chronic Kidney Disease Patients and Its Association With Serum Free Immunoglobulin Light Chains

**DOI:** 10.3389/fimmu.2021.609700

**Published:** 2021-04-01

**Authors:** Fengping Liu, Xuefang Xu, Lin Chao, Ke Chen, Amo Shao, Danqin Sun, Yan Hong, Renjing Hu, Peng Jiang, Nan Zhang, Yonghong Xiao, Feng Yan, Ninghan Feng

**Affiliations:** ^1^ Wuxi School of Medicine, Jiangnan University, Wuxi, China; ^2^ Department of Urology, Affiliated Wuxi No. 2 Hospital, Nanjing Medical University, Wuxi, China; ^3^ Department of Nephrology, Affiliated Wuxi No. 2 Hospital, Nanjing Medical University, Wuxi, China; ^4^ Department of Thyroid and Breast, Affiliated Wuxi No. 2 Hospital, Nanjing Medical University, Wuxi, China; ^5^ Wuxi Higher Health School, Wuxi, China; ^6^ Department of Laboratory Medicine, Affiliated Wuxi No. 2 Hospital, Nanjing Medical University, Wuxi, China; ^7^ Collaborative Innovation Center for Diagnosis and Treatment of Infectious Diseases, State Key Laboratory for Diagnosis and Treatment of Infectious Diseases, The First Affiliated Hospital, School of Medicine, Zhejiang University, Hangzhou, China

**Keywords:** *Bifidobacterium*, chronic kidney disease, free immunoglobulin light chains, gut microbiome, confounders

## Abstract

**Objectives:**

Gut dysbiosis is associated with chronic kidney disease (CKD), and serum free immunoglobulin light chains (FLCs) are biomarkers for CKD. This study aims to assess the CKD gut microbiome and to determine its impact on serum FLC levels.

**Methods:**

To control for confounders, 100 patients and sex- and age-matched healthy controls (HCs) were recruited. The gut microbiome was assessed by sequencing 16S rRNA gene V3-V4 hypervariable regions. Phylogenetic Investigation of Communities by Reconstruction of Unobserved States was applied to infer functional metabolic pathways. When observing group differences in the microbiome and predicted metabolic pathways, demographic confounders were adjusted using binary logistic regression; when examining impacts of the gut microbiome and metabolic pathways on serum FLCs, factors influencing FLC levels were adjusted using multiple regression.

**Results:**

Principal coordinate analysis revealed a significantly different bacterial community between the CKD and HC groups (P < 0.05). After adjusting for confounders, lower Chao 1, observed species and Shannon indices based on binary logistic regression predicted CKD prevalence. Actinobacteria, Alistipes, Bifidobacterium and Bifidobacterium longum enrichment, upregulation of metabolic pathways of bacterial toxin, chloroalkane and chloroalkene degradation, and Staphylococcus aureus infection also predicted CKD prevalence (P < 0.05). Furthermore, depletion of Actinobacteria and Bifidobacterium and reduced chloroalkane and chloroalkene degradation predicted high levels of FLC λ (P < 0.05).

**Conclusions:**

Gut dysbiosis in CKD patients was confirmed by controlling for confounders in the present study. Additionally, the association between gut dysbiosis and FLC λ levels demonstrates the existence of crosstalk between the microbiome and immune response in CKD.

## Introduction

Chronic kidney disease (CKD) is considered a major global health problem and is characterized by gradually declining kidney function. Evidence indicates that the consequences of kidney injury may originate from alterations in the gut microbial population (1). For example, Vaziri et al. demonstrated extensive changes in the bacterial community and function of the gut microbiome in patients with end-stage renal disease (ESRD) on hemodialysis . The bacterial families of Actinobacteria, Firmicutes, and Proteobacteria were shown in one study to have the largest increases in patients with ESRD compared with healthy controls (1). Wang IK et al. found that patients on peritoneal dialysis compared with healthy subjects (2). Patients on peritoneal dialysis were less likely to have *Bifidobacterium catenulatum*, *B. longum*, *B. bifidum*, *Lactobacillus plantarum*, *L. paracasei*, and *Klebsiella pneumoniae* (2). Wong J et al. Demonstrated that ESRD patients maintained on hemodialysis exhibited significant expansion of bacterial families possessing urease, uricase, and indole and p-cresol forming enzymes, and contraction of families possessing butyrate-forming enzymes (3). As can be seen from the abovementioned studies, almost the previous studies were focused on patients who had undergone dialysis. However, Ren et al. characterized the gut microbiome in Chinese CKD population who had never been on dialysis and CKD-related drug therapy illustrated that the microbial community in the patient cohort was different from this in the healthy subjects. In addition, when the bacterial genus level was compared using Wilcoxon rank-sum test, LEfSe analysis and receiver operating characteristic curve, the authors also found significant difference between the two cohorts. For example, Klebsiella and Enterobacteriaceae increased, while Blautia and Roseburia decreased in patient cohort (4).

In addition, emerging studies have reported that gut dysbiosis contributes to the disruption in intestinal barrier function in CKD, allowing the translocation of gut-derived toxins, bacterial products, and intact bacteria into the circulation (5, 6). In other words, gut dysbiosis may contribute to the accumulation of uremic toxins in patients with CKD (7).

Uremic toxins, retention solutes that accumulate in the serum of patients with reduced kidney function, contribute to a variety of metabolic and functional disturbances, such as reduced immunological defense. Free immunoglobulin light chains (FLCs), including FLC κ and λ, are the small polypeptide subunits of an antibody. FLCs act as uremic toxins by interfering with the essential function of polymorphonuclear leukocytes (8). FLCs can inhibit neutrophil apoptosis; moreover, interfering with the resolution of inflammation may perpetuate a chronic inflammatory state, which has been shown to be associated with adverse outcomes in patients with CKD (8–10). Not only an elevation of FLCs is a significant predictor of worse overall survival in the general population (11), but also both serum and urinary FLCs can predict the CKD stage and the severity of albuminuria (12), and the ratio of FLC κ/creatinine and FLC λ/creatinine was negatively associated to patients’ eGFR and elevated with patients’ CKD stages (13).

FLCs activate mast cells, which may accelerate both atherosclerosis and myocardial fibrosis and can contribute to the development of interstitial fibrosis in the kidney (14). FLCs clearance is dependent on glomerular filtration, and serum FLCs increase as kidney function declines (14). Furthermore. FLCs are associated with the risk of end-stage renal disease and death in CKD patients (15). Thus, it is critical to investigate factors associated with the elevation of serum FLCs.

As pathogenic alterations in commensal microorganisms contribute to disease manifestations through effects on host immunity (16), we aimed to compare the microbial profile of patients with CKD and healthy controls (HC). In addition, we aimed to assess putative associations between the microbiome and FLCs levels, which might provide clues for the treatment of CKD.

## Methods

### Patient

The ethics committee of the Affiliated Wuxi Second Hospital of Nanjing Medical University approved this study (Ref. 2018051). Informed consent was provided by all subjects prior to sample collection. Based on the sample size calculation method for the case-control human microbiome study created by Mattiello et al. (https://fedematt.shinyapps.io/shinyMB/) (17), ninety-two subjects were required in each group to achieve a power of 0.80. Therefore, 100 adult CKD patients and 100 HCs were recruited in the present study from December 2018 to February 2020. Age and sex were matched to control for confounding variables (18). All participants, including the CKD patients and healthy subjects, were local residents.

CKD involves two clinical conditions: either of kidney damage or eGFR < 60 ml/min/1.73 m^2^ present for ≥ 3 months (18). Kidney damage refers to pathologic abnormalities or markers of damage, including abnormalities in blood or urine tests or imaging studies (19). The markers of kidney damage include albuminuria [(albumin excretion rate ≥ 30 mg/24 h; urinary albumin creatinine ratio (UACR) ≥ 30 mg/g], urine sediment abnormalities, electrolyte and other abnormalities due to tubular disorders, abnormalities detected by histology, and structural abnormalities detected by imaging (20). In clinical practice, this means that in order to diagnose CKD in an individual with normal eGFR or with eGFR ≥ 60 mL/min/1.73 m^2^ (21). Patients with CKD who had never undergone hemodialysis were recruited. Subjects with kidney damage (the markers of kidney damage as described above) or eGFR above 90 ml/min/1.73 m^2^, current illness, acute or chronic infections, elevated body temperature, white blood cell count or CRP were excluded from the HC group. In addition, participants with acute intercurrent disease and infections, diarrhea, kidney transplantation, pregnancy, and breastfeeding and those who used antibiotics, probiotic or immunosuppressive drugs within one month before enrollment were excluded from the present study.

An immunoturbidimetric test was used to assess serum levels of polyclonal FLC κ and λ on the day of sample collection by Freelite test (AU5421; Beckman Coulter, USA). FLC κ and λ test kits were purchased from Jingyuan Co., Ltd (Lot number: 20152400201; Shanghai, China). In our lab, the normal reference range of 2.0 to 4.4 g/L for serum FLC κ and 1.1 to 2.4 g/L for serum FLC λ was used, and Freelite result of 0.26-1.65 was the reference range for serum FLC κ/λ ratio (22). The normal reference range of 0-18.5 mg/L for urinary FLC κ and 0-50 mg/L for urinary FLC λ was used. Information on clinical manifestations was obtained by reviewing clinical records. In addition, concurrent diseases, such as diabetes, hypertension and hyperlipidemia, and current medication usage were assessed by reviewing clinical records, medical interviews and routine check-ups.

### Sample Collection and DNA Isolation

Fresh fecal material was collected in a sterile container, and 30 mg was placed in another sterile container. All samples were immediately stored at −80°C until further processing. DNeasy PowerSoil Pro Kit was used to isolate microbial genomic DNA from fecal samples (QIAGEN, Germany), and the isolation procedures were performed according to the manufacturer’s instructions. The total DNA was eluted in 50 μl of elution buffer and stored at −80°C until used for PCR. PCR products were confirmed by 2% agarose gel electrophoresis. Throughout the DNA extraction process, ultrapure water, instead of a sample solution, was used as a negative control to exclude the possibility of false-positive PCR results. Polymerase chain reaction (PCR) amplification of the bacterial 16S rRNA genes V3-V4 region was performed using the universal primers 338F and 806R with 30 cycles. The PCR products were purified with AMPure XT beads (Beckman Coulter Genomics, Danvers, MA, USA) and quantified by Qubit (Invitrogen, Waltham, MA, USA). Amplicon pools were prepared for sequencing, and the size and quantity of the amplicon library were assessed using an Agilent 2100 Bioanalyzer (Agilent, Santa Clara, MA, USA) and Library Quantification Kit for Illumina (Kapa Biosciences, Woburn, MA, USA), respectively. The libraries were sequenced using the NovaSeq PE250 platform.

### Bioinformatic Analysis

Paired-end reads were assigned to samples based on their unique barcodes and truncated by cutting off the barcode and primer sequence. Paired-end reads were merged using FLASH. Quality filtering of the raw reads was performed under specific filtering conditions to obtain high-quality clean tags according to fqtrim (v. 0.94). Chimeric sequences were filtered using Vsearch software (v. 2.3.4). After dereplication using DADA 2, we obtained a feature table and feature sequence. Alpha diversity and beta diversity were calculated by normalization to the same sequences randomly using QIIME2 (23). Next, according to the SILVA (v. 132) classifier (24), feature abundance was normalized using the relative abundance of each sample. Alpha diversity was applied to analyze the complexity of amplicon sequence variant (ASV) for a sample through the Chao1, observed species, Shannon and Simpson indices. Beta diversity analysis was performed to evaluate differences in species complexity between samples. We applied permutational multivariate analysis of the variance method to Bray–Curtis distance data using 999 permutations to examine feature differences between patients with CKD and HCs; statistical significance was defined as *P* < 0.05 (R software vegan package). Blast was used for sequence alignment, and the feature sequences were annotated with the SILVA database. Phylogenetic Investigation of Communities by Reconstruction of Unobserved States (PICRUSt) analysis was performed to identify Kyoto Encyclopedia of Genes and Genomes (KEGG) metabolic pathways potentially affected by groups of bacteria.

The sequencing data obtained in this study have been deposited in GenBank Sequence Read Archive under accession number SRP279052 (https://www.ncbi.nlm.nih.gov/sra?term=SRP279052&cmd=DetailsSearch).

### Statistical Methods

Descriptive statistics for demographics and clinical characteristics of the CKD and HC groups are presented. To compare demographics and clinical characteristics between the CKD and HC groups, continuous variables were assessed using independent *t*-tests and categorical variables using chi-square or Fisher’s exact tests, when appropriate. One-way analysis of variance was employed to compare quantitative variables among CKD subgroups.

The Wilcoxon rank sum test was applied to compare bacterial diversity and the relative abundance of bacterial taxa and metabolic pathways between the CKD and HC groups, and a Benjamini Hochberg false discovery rate (FDR)-corrected *P*-value was calculated for comparative tests. A *P*-value < 0.05 was used as a cut-off for comparative statistical tests. To control for potential confounders, we performed binary logistic regression analysis with SPSS (v. 24.0) to adjust for covariates when they presented significant differences between the CKD and HC groups.

Consequently, we examined associations between the abundance of bacterial taxa and metabolic pathways that exhibited significant differences between the CKD and HC groups and levels of serum FLC κ and λ. In association analysis, we used multiple linear analysis with SPSS (v. 24.0), which can adjust for potential confounding factor(s) that impact the level of FLC κ or λ. A *P*-value < 0.05 was used as significance cut-off regression analysis and linear analysis.

## Results

### Demographics

As **Table 1** shows, compared to the HC group, the CKD patients had significantly higher BMI, C-reactive protein (CRP), eGFR, serum urea, serum creatinine, FLC κ and λ and higher rates of positive urine protein, hypertension and hyperlipidemia (*P* < 0.05). Although the patient cohort had slightly higher level of serum FLC κ/λ ratio comparing to the HC cohort (*P* > 0.05), the alteration was in normal ranges (25). Interestingly, when the CKD patients were stratified as CKD stages, we noticed that only the levels of eGFR and serum uric acid differed among them (*P* < 0.05).

**Table 1:** Characteristics of the participants.

Parameters	CKD (n = 100)	HC (n = 100)	p value
Age, yr	56.64 ± 17.25	60.64 ± 16.51	0.101
Men	50 (50)	50 (50)	1.000
Body mass index (kg/m^2^)	25.08 ± 3.59	23.62 ± 2.75	0.002
Body temperature (°C)	36.68 ± 0.26	36.58 ± 0.34	0.058
C-reactive protein (mg/L)	5.63 ± 10.28	1.75 ± 1.17	0.000
White blood cell count (billion cells/L)	6.39 ± 1.53	6.04 ± 3.73	0.429
eGFR (mL/min/1.73m^2^)	56.95 ± 39.27	100.90 ± 9.41	<0.001
Serum urea (mmol/L)	13.11 ± 11.96	3.22 ± 2.76	<0.001
Serum creatinine (mg/dL)	185.24 ± 180.36	30.63 ± 29.46	<0.001
Urine protein			<0.001
Negative	15	100	<0.001
Positive 1 plus	48	0	<0.001
Positive 2 plus	30	0	<0.001
Positive 3 plus	7	0	<0.001
Serum FLC κ (g/L)	8.99 ± 2.98	2.83 ± 0.55	<0.001
Serum FLC λ (g/L)	4.80 ± 1.53	1.75 ± 0.29	<0.001
Serum κ/λ ratio	2.01 ± 1.28	1.63 ± 0.29	0.313
Urinary FLC κ (mg/L)			NA
0-18.5 mg/L	25	NA	
≥ 18.5 mg/L	75	NA	
Urinary FLC λ (mg/L)			NA
0-50 mg/L	71	NA	
≥ 50 mg/L	29	NA	
CKD stage			
Stage 1	24	NA	NA
Stage 2	20	NA	NA
Stage 3	20	NA	NA
Stage 4	18	NA	NA
Stage 5	18	NA	NA
Co-current disease			
Hypertension	76	32	0.000
Type 2 diabetes mellitus	29	18	0.067
Hyperlipidemia	29	14	0.017
Medication usage			
Antihypertensive agent	63	15	<0.001
Metformin	19	8	0.023
Antilipemic agent	41	NA	NA
Diuretics	14	NA	NA
Platelet aggregation inhibitor	15	NA	NA
Antihyperuricemic agent	13	NA	NA
Folic acid supplement	18	NA	NA
Calcium supplement	22	NA	NA

Pearson’s Chi-square/Fisher’s exact test was used to compare dichotomous variables, and an independent *t*-test was used to compare continuous variables. NA, not applicable.

### Bacterial Community in Participants

In total, 15,345,951 raw reads were obtained (average raw reads were 79,102; ranging from 49,121 to 122,701); 12,926,322 reads were obtained after removing low-quality or ambiguous reads. Good coverage ranged from 99.42% to 100.00%.

**Figure 1:**
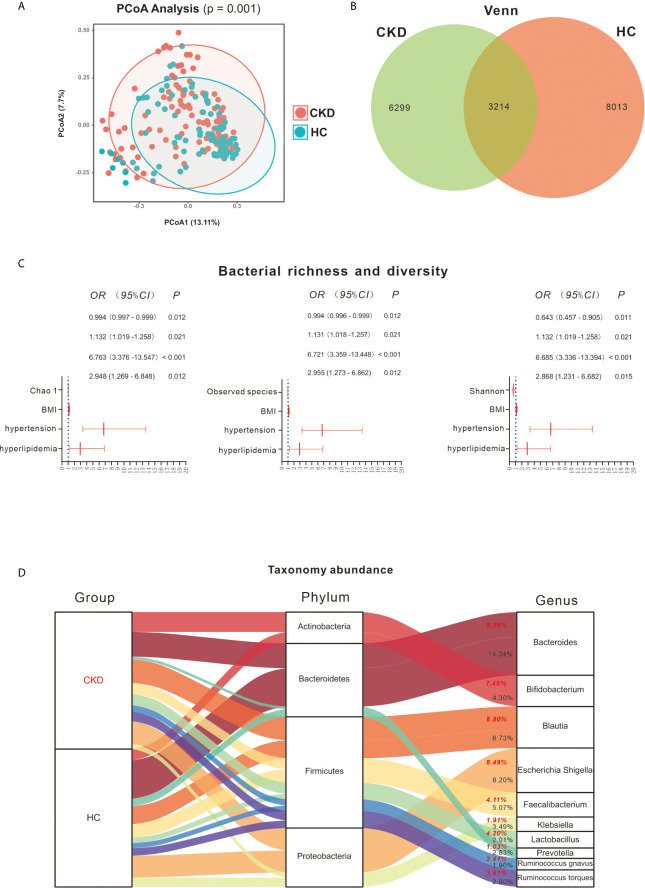
Bacterial community, diversity and profile. **(A)** Principal coordinates analysis (PCoA) revealed clustering of bacterial taxa in the CKD and HC groups based on Bray–Curtis distance, with each point corresponding to a subject and colored according to the sample type. Permutational multivariate analysis of variance showed that the separation of bacterial communities in the CKD and HC cohort was significant (*P* = 0.001). **(B)** Venn diagram showing the shared number of operational taxonomic units by CKD and HC subjects. **(C)** Bacterial richness and diversity between the CKD and HC cohorts. Binary regression analysis was used to adjust for confounders of BMI, hypertension and hyperlipidemia. The estimated ORs and their CIs are displayed as forest graphs. The blue dotted line represents the OR value = 1. Comparison of gut microbiome richness and diversity shows that lower Chao 1, observed species and Shannon indices can predict the prevalence of CKD (*P* < 0.05). **(D)** Bacterial profile in the CKD and HC groups. Red font represents the CKD group and the bacterial abundance in this group; black font represents the HC group and the bacterial abundance in this group.

PCoA revealed a significant difference in bacterial composition between the CKD and HC groups (**Figure 1A**; *P* = 0.001). However, when the patients were separated into subgroups based on CKD stage, no significant difference between the subgroups was observed (**Figure S1**; *P* > 0.05). The Venn diagram in **Figure 1B** illustrates 9,513 and 11,227 observed features in the CKD and HC samples, respectively, of which 3214 (18.34%) were shared.

As significant differences between the CKD and HC groups were found for the demographics of BMI, co-occurrence of hypertension and hyperlipidemia (**Table 1**), they were considered as potential confounders when comparing differences in bacterial diversity, taxa and metabolic pathways between the groups.

Based on Wilcoxon rank analysis, all of the bacterial richness and diversity indices were significantly decreased in the CKD group compared to the HC group (data not shown; *P* < 0.05). However, when adjusting for the abovementioned confounders, only Chao 1 [odds ratio (OR) = 0.994; 95% confidence interval (CI) 0.997, 0.999], observed species (OR = 0.994; 95% CI 0.996, 0.999) and Shannon (OR = 0.643; 95% CI 0.457, 0.905) index reductions were significantly associated with the CKD group (*P* < 0.05, **Figure 1C**).

### Bacterial Profile in the CKD and HC Groups

As shown in **Figure 1D**, Firmicutes, Bacteroidetes, Proteobacteria and Actinobacteria were the most abundant bacterial phyla in the participants, accounting for 98.35% and 98.58% in the CKD and HC groups, respectively. Firmicutes taxa mainly involved *Blautia* (8.80% and 6.73% in CKD and HC, respectively), *Faecalibacterium* (4.11% and 5.07% in CKD and HC, respectively), *Lactobacillus* (4.20% and 2.01% in CKD and HC, respectively), *Ruminococcus gnavus* (2.41% and 1.90% in CKD and HC, respectively), and *Ruminococcus torques* (3.61% and 2.80%, respectively). For Bacteroidetes, *Bacteroides* (9.26% and 14.34% in CKD and HC, respectively) and *Prevotella* (1.03% and 2.83% in CKD and HC, respectively) were mainly detected. *Escherichia, Shigella* (8.49% and 8.20% in CKD and HC, respectively) and *Klebsiella* (1.91% and 3.49% in CKD and HC, respectively) mostly accounted for Proteobacteria and *Bifidobacterium* (7.45% and 4.30% in CKD and HC, respectively) for Actinobacteria.

**Figure 2:**
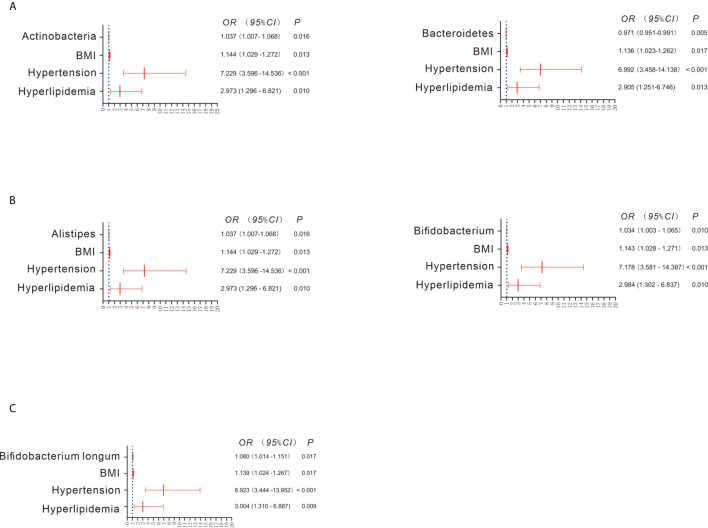
Bacterial abundance showing a significant difference between the CKD and HC groups when adjusting for confounders **(A–C)**. Binary regression analysis was used to adjust for confounders of BMI, hypertension and hyperlipidemia. The estimated ORs and their CIs are displayed as forest graphs. The blue dotted line represents the OR value = 1.

The Wilcoxon rank test showed 6 bacterial phyla exhibiting significant differences between the CKD and HC groups (**Figure S2A**). Among them, Actinobacteria was significantly increased in the CKD group compared to the HC group, whereas Bacteroidetes was significantly reduced in the CKD group with respect to the HC group. Similar to the findings using the Wilcoxon rank test, after adjustment for the confounders BMI, hypertension and hyperlipidemia, the binary regression model showed that an increase in Actinobacteria predicted CKD prevalence (OR = 1.037; 95% CI 1.007, 1.068; **Figure 2A**). In addition, a decrease in Bacteroidetes predicted the prevalence of CKD (OR = 0.971; 95% CI 0.951, 0.991). However, the remaining five bacteria did not predict the prevalence of CKD when adjusting for confounders.

When the bacterial genera were compared using the Wilcoxon rank test, significant differences between the CKD and HC groups were found for 41 taxa. For example, *Bifidobacterium*, *Enterococcus*, and *Streptococcus* were significantly increased in the CKD group, whereas *Alistipes*, *Bacteroides*, and *Prevotella* were significantly decreased in the CKD group (**Figure S2B**). Interestingly, after adjusting for confounders, only the increase in *Alistipes* (OR = 1.037; 95% CI 1.007, 1.068) and *Bifidobacterium* (OR = 1.034; 95% CI 1.003, 1.065) was associated with CKD prevalence (**Figure 2B**). As depicted in **Figure S2C**, all bacterial species exhibiting significant differences were more abundant in the CKD group than in the HC group, such as *Bifidobacterium longum*, *Faecalibacterium prausnitzii* and *Lactobacillus crispatus*. However, when adjusting for confounders, only a higher abundance of *Bifidobacterium longum* predicted the prevalence of CKD (OR = 1.080; 95% CI 1.014, 1.151; **Figure 2C**).

### Metabolic Pathway Differences Between the CKD and HC Groups

**Figure 3:**
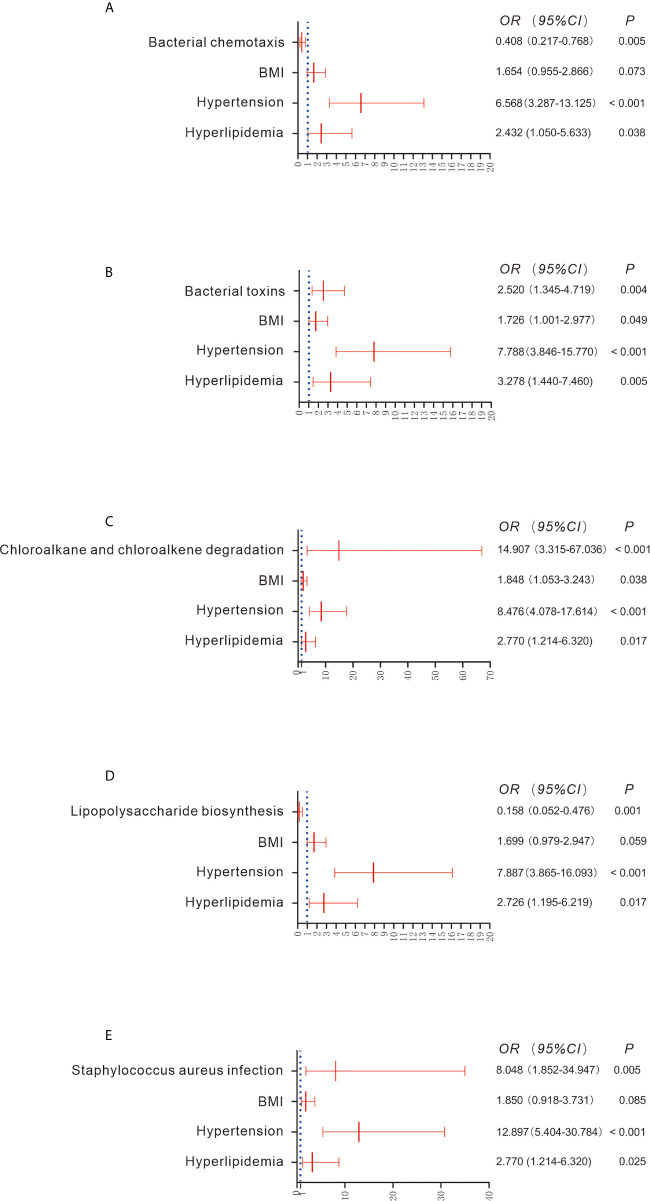
Comparison of functional pathways between the CKD and HC groups **(A–E)**. Gene functions were predicted based on 16S rRNA gene-based microbial compositions using the PICRUSt algorithm and the Kyoto Encyclopedia of Genes and Genomes database. Binary regression analysis was used to adjust for confounders of BMI, hypertension and hyperlipidemia. The estimated ORs and their CIs are displayed as forest graphs. The blue dotted line represents the OR value = 1.

There were 61 metabolic pathways that exhibited significant differences between the CKD and HC groups when compared using the Wilcoxon rank test (**Table S1**; *P* < 0.05), though only 5 pathways were significantly associated with the prevalence of CKD after adjustment for the confounders BMI, co-occurrence of hypertension and hyperlipidemia (*P* < 0.05; **Figures 3A–E**). For example, increased bacterial toxins (OR = 2.520; 95% CI 1.345, 4.719), chloroalkane and chloroalkene degradation (OR = 14.907; 95% CI 3.315, 67.036) and *Staphylococcus aureus* infection (OR = 8.048; 95% CI 1.852, 34.947) predicted the prevalence of CKD. Downregulation of bacterial chemotaxis (OR = 0.408; 95% CI 0.217, 0.768) and lipopolysaccharide biosynthesis (OR = 0.158; 95% CI 0.052, 0.476) also predicted the prevalence of CKD (**Figure 3**).

### Gut Microbiome Predicted Serum FLC Levels

When the CKD patients were stratified by sex, age, BMI, stage of CKD, concurrent diseases and medication usage, we observed that the serum levels of FLC κ and λ were significantly different in those with and without hyperlipidemia (7.57± 2.37 vs. 9.54 ± 3.02, *P* = 0.003; 4.11 ± 1.46 vs. 5.06 ± 1.49, *P* = 0.005). Thus, hyperlipidemia was considered a covariate when performing multiple linear regression analysis to evaluate the influence of bacterial taxa and metabolic pathways on the levels of FLC κ and λ. After confounder adjustment, the abundance of bacterial taxa or metabolic pathways that showed significant differences between the CKD and HC groups did not affect the levels of serum FLC κ (data not shown; *P* > 0.05). In other words, the CKD-enriched or CKD-depleted microbiota/metabolic pathways were selected to perform the correlation analysis. Interestingly, we observed that a decline in the abundance of Actinobacteria and *Bifidobacterium* predicted increased levels of FLC λ (*P* < 0.05, **Tables 2** and **3**), and a decline in chloroalkane and chloroalkene degradation also predicted increased levels of FLC λ (**Table 4**, *P* < 0.05).

**Table 2:** Linear regression analysis for Actinobacteria predicting FLC λ.

Variable	*B*	*SE*	*β*	*t*	*p*	VIF
Constant	5.269	0.198		26.599	< 0.005	
Actinobacteria	-0.022	0.011	-0.198	-2.061	0.042	1.009
Hyperlipidemia	-0.891	0.326	-0.262	-2.735	0.007	1.009
Non-Hyperlipidemia	0					
*R* ^2^		0.118				
*F*		6.460				
*P*		0.002				

Multiple linear analysis was used to assess the association between the abundance of Actinobacteria and serum FLC λ concentration, with adjustment for the confounder hyperlipidemia. In the analysis, hyperlipidemia was treated as a dichotomous variable.

**Table 3:** Linear regression analysis for *Bifidobacterium* predicting FLC λ.

Variable	*B*	*SE*	*β*	*t*	*p*	VIF
Constant	5.222	0.185		28.224	< 0.005	
Bifidobacterium	-0.024	0.011	-0.212	-2.225	0.028	1.008
Hyperlipidemia	-0.889	0.324	-0.262	-2.741	0.007	1.008
Non-Hyperlipidemia	0					
*R* ^2^	0.124					
*F*	6.840					
*P*	0.002					

Multiple linear analysis was used to assess the association between the abundance of *Bifidobacterium* and serum FLC λ concentration, with adjustment for the confounder hyperlipidemia. In the analysis, hyperlipidemia was treated as a dichotomous variable.

**Table 4:** Linear regression analysis for chloroalkane and chloroalkene degradation predicting FLC λ.

Variable	*B*	*SE*	*β*	*t*	*p*	VIF
Constant	6.998	0.995		7.328	< 0.005	
Chloroalkane and chloroalkene degradation	-9.391	4.564	-0.197	-2.058	0.042	1.002
Hyperlipidemia	-0.922	0.325	-0.271	-2.841	0.005	1.008
Non-Hyperlipidemia	0					
*R* ^2^	0.117					
*F*	6.453					
*P*	0.002					

Multiple linear analysis was used to assess the association between chloroalkane and chloroalkene degradation and the serum FLC λ concentration, with adjustment for the confounder hyperlipidemia. In the analysis, hyperlipidemia was treated as a dichotomous variable.

## Discussion

A recent previous study reported that Chinese CKD patients have altered gut microbiota comparing to HC cohort (4). In the previous study, the patients on dialysis also excluded. In addition, the healthy subject with hypertension and diabetes were excluded, which can rule out the influence of co-current diseases on the gut microbiota (4). Based on the prevalence of CKD and its common complications of hypertension and diabetes, the CKD patients with hypertension and diabetes, and the non-CKD healthy subjects with hypertension and diabetes were included in our present study. As hypertension and diabetes are common complications in CKD patients (26), and CKD is common in the elderly (27), it is hard to recruit healthy subjects who are age matched with CKD patients in clinical settings. When the gut microbiota was compared between CKD patients and HCs, the confounders such as BMI and comorbid diseases were adjusted in present study. As we excluded dialysis patients, there was no confounding due to effects of dialysis on the microbiota (28). Potential impact of inflammation on the HC microbiome was minimized by excluding HCs with infection or elevated inflammatory markers (CRP, white cell count, body temperature) (28). Overall, our study illustrates that gut dysbiosis and metabolic pathways can predict levels of serum FLC λ in CKD patients after adjustment for hyperlipidemia.

CKD patients in the present study had lower bacterial diversity compared to HC subjects and different bacterial communities, which was consistent with a previous study on the Chinese population (29). Interestingly, no significant differences in the bacterial community in patients at different CKD stages were found, which has not been reported in previous studies. Similar to the gut microbiome composition in CKD stages, the levels of FLC κ and λ did not exhibit significant difference among the five stages. The alterations of FLC κ and λ were dissimilar to the findings reported by Desjardins L et al. (30). In their study, FLC κ and λ levels rose progressively with the CKD stage (30). The dissimilarity between the two studies might be due to the patients in our study had not undergone dialysis at recruitment. It is demonstrated that hemodialysis contributes to serum FLCs (30). Additionally, the different FLCs detection methods might be responsible for the inconsistent findings of Desjardins L and ours, as FLCs are different by Freelite assay and by N Latex assay (31). The non-synchronicity of the alterations between microbiome composition and CKD stage and between microbiome composition and FLC κ/λ indicate that the rate of loss in kidney function decline appear to be inconsistent with the alterations of gut microbiome and FLCs.

An increase in Actinobacteria in CKD patients has been observed by Vaziri et al. and Li F et al. (1, 29). In the Vaziri et al. study, the increase in Actinobacteria was attributed to the abundance of the bacterial families Brachybacterium and Nesterenkonia in CKD patients (1). However, in our study, the high abundance of the bacterial genus *Bifidobacterium* and *Bifidobacterium longum* in CKD patients was attributed to the increase in Actinobacteria, which has not been reported before. Recently, particular interest has focused on members of *Bifidobacterium*, which have been included as live components in a variety of so-called probiotics to recover kidney function in CKD patients. For example, probiotics containing *B. longum* can lower blood urea nitrogen, uric acid, uremic toxin and inflammatory marker concentrations in CKD patients (32–34). Similar to human studies, Iwashita Y et al. and Wang X et al. demonstrated that *Bifidobacterium* or *B. longum* can lower serum toxins in CKD mice (35, 36). Thus, the remarkable increase in *Bifidobacterium* and *B. longum* in the gut of CKD patients may have a role in slowing CKD progression. It is worth noting that a decrease in *Bifidobacterium* predicted an increase in serum FLC λ in CKD patients in the present study. FLCs are rapidly cleared from the serum and are largely filtered by the kidneys; accordingly, the kidneys are a prominent target for FLC deposition and often sustain damaged. For example, Erdem BK and coauthors reported that FLC λ can be considered a useful marker for predicting renal function (37). The negative association between *Bifidobacterium* and FLC λ indicates that this genus is responsible for alleviating the damage to kidneys caused by serum FLC λ accumulation. This association also suggests that elevation of *Bifidobacterium* in the gut is a protective response in CKD patients.

We observed that Bacteroidetes depletion can predict the prevalence of CKD. In a previous study on a Chinese population, Jiang S et al. reported a similar trend in CKD patients compared to HC subjects (38). Interestingly, we noticed that enrichment of *Alistipes*, a bacterial genus in Bacteroidetes, can predict CKD prevalence. *Alistipes* has been considered an ESRD-enriched bacterium that can result in higher levels of serum uremic toxins in patients, according to Wang X et al. (36); thus, overgrowth of *Alistipes* in CKD might be a pathologic factor deteriorating the patient’s condition.

In the present study, several metabolic pathways were enriched in the CKD group, which has not been observed in previous studies. For instance, we noted that enrichment of bacterial toxin pathways and *Staphylococcus aureus* infection are related to the prevalence of CKD. Bacterial toxins can induce activation of the NLRP3 inflammasome (39), and *S. aureus* can impact the effectiveness of the human immune system, such as secreting immune modulating proteins to inhibit complement activation (40). Therefore, enrichment of bacterial toxins and *S. aureus* infection might be responsible for the high prevalence of various infections in CKD patients. Interestingly, we also found that the increase in chloroalkane and chloroalkene degradation pathway predicted CKD prevalence. Chloroalkane and chloroalkene degradation is one of the pathways in xenobiotic biodegradation and metabolism. The kidneys have a defined role as excretory organs for xenobiotics in the circulation, and the pathway for xenobiotic elimination depends on glomerular filtration (41). Thus, upregulation of chloroalkane and chloroalkene degradation might be due to loss of kidney function. In the present study, a high level of chloroalkane and chloroalkene degradation pathway predicted a low level of serum FLC λ, suggesting the existence of similar mechanisms in the kidneys to clear chloroalkane and FLC λ. Notably, although lipopolysaccharide is the major component of the outer membrane of gram-negative bacteria, consisting of three domains that are involved in toxicity and pathogenicity (42), the pathway of lipopolysaccharide biosynthesis was downregulated in the CKD group. This finding might be because the patients were not infected at recruitment, and lipopolysaccharide is frequently accompanied by various infections (43–45).

It is worth noting that our present study only found that the microbiome was associated to the level of serum FLC λ, but not FLC κ. This might be due to the alteration of FLC λ is not consistent with FLC κ. Similar phenomenon was observed in healthy adults completed marathon running (46), which may induce acute and transient renal impairment (47). Further study is needed to explore why and how gut microbiome stimulate FLC λ.

One limitation of the present study is that the duration of recruitment of the participants lasted for over one year, during which we could not rule out seasonal factors associated with the microbiome. Second, we only collected gut samples from local residents, which can only illustrate alteration in the gut microbiome of local CKD patients. Participants from multiple locations should be included in future studies. Thirdly, the numbers of subjects per CKD stage and comorbid conditions were rather small in the present study. Although they were listed as confounders and adjusted in the statistical analysis, it is possible influence the findings. Last but not least, PICRUSt was used to infer functional gene and pathway profiles from 16S rRNA gene data in the present study (48–52), the associations between microbiome and functional pathways, between microbiome and FLCs, and between functional pathways and FLC λ should be confirmed using fecal transplantation in animal experiment and human fecal metabonomics.

## Conclusions

The CKD patients in this study exhibited gut dysbiosis after controlling for confounders, which may account, in part, for the increased levels of serum FLC λ in these patients. The results highlight the need to utilize microbiome-based interventions to eliminate bacterial toxins in CKD patients, including FLC λ.

## Data Availability Statement

The datasets presented in this study can be found in online repositories. The names of the repository/repositories and accession number(s) can be found below: https://www.ncbi.nlm.nih.gov/genbank/, SRP279052.

## Ethics Statement

The ethics committee of the Affiliated Wuxi Second Hospital of Nanjing Medical University approved this study (Ref. 2018051). The patients/participants provided their written informed consent to participate in this study.

## Author Contributions

Study design: NF, FY, YX, and FL. Literature search: AS and FL. Methodology: LC, KC, XX, NZ, PJ, AS, DS, YH, RH, and FY. Software: FL. Validation: FL and NF. Formal analysis: FL. Investigation: NZ and PJ. Resources: NF, LC, and FY. Data interpretation: FL, NZ, and PJ. Writing—original draft preparation: FL. Writing—review and editing: FL, NF, and YX. Visualization: FL. Supervision: FL and NF. Project administration: NF and FL;. Funding acquisition: NF. All authors contributed to the article and approved the submitted version.

## Funding

This research was funded by the Special Project of Medical Innovation Team in Jiangsu Province (grant number: CXTDA2017047). The funding body had no role in the design of the study, the collection, analysis, or interpretation of the data or in the writing of the manuscript.

## Conflict of Interest

The authors declare that the research was conducted in the absence of any commercial or financial relationships that could be construed as a potential conflict of interest.

## Supplementary Material

Supplementary Figure 1 Principal coordinates analysis (PCoA) revealed clustering of bacterial taxa in the six stages of CKD based on Bray–Curtis distance, with each point corresponding to a subject and colored according to the sample type. Permutational multivariate analysis of variance showed that the separation of bacterial communities in the six stages was not significant (*P* > 0.05).

Supplementary Figure 2 Bacterial taxon abundance showing a significant difference between the CKD and HC groups without adjusting confounders (*P* < 0.05). The Wilcoxon rank-sum test was used to compare differences in abundance between the two groups.

## References

[1] Vaziri ND Wong J Pahl M Piceno YM Yuan J DeSantis TZ. Chronic kidney disease alters intestinal microbial flora. Kidney Int (2013) 83:308–15. 10.1038/ki.2012.345 22992469

[2] Wang IK Lai HC Yu CJ Liang CC Chang CT Kuo HL. Real-time PCR analysis of the intestinal microbiotas in peritoneal dialysis patients. Appl Environ Microbiol (2012) 78:1107–12. 10.1128/AEM.05605-11 PMC3273023 22179250

[3] Wong J Piceno YM DeSantis TZ Pahl M Andersen GL Vaziri ND. Expansion of urease- and uricase-containing, indole- and p-cresol-forming and contraction of short-chain fatty acid-producing intestinal microbiota in ESRD. Am J Nephrol (2014) 39:230–7. 10.1159/000360010 PMC4049264 24643131

[4] Ren Z Fan Y Li A Shen Q Wu J Ren L. Alterations of the Human Gut Microbiome in Chronic Kidney Disease. Adv Sci (Weinh) (2020) 7:2001936. 10.1002/advs.202001936 33101877 PMC7578882

[5] Brenchley JM Douek DC. Microbial translocation across the GI tract. Annu Rev Immunol (2012) 30:149–73. 10.1146/annurev-immunol-020711-075001 PMC3513328 22224779

[6] Vaziri ND Zhao YY Pahl MV. Altered intestinal microbial flora and impaired epithelial barrier structure and function in CKD: the nature, mechanisms, consequences and potential treatment. Nephrol Dial Transplant (2016) 31:737–46. 10.1093/ndt/gfv095 25883197

[7] Ramezani A Raj DS. The gut microbiome, kidney disease, and targeted interventions. J Am Soc Nephrol (2014) 25:657–70. 10.1681/ASN.2013080905 PMC3968507 24231662

[8] Cohen G Haag-Weber M Mai B Deicher R Horl WH. Effect of immunoglobulin light chains from hemodialysis and continuous ambulatory peritoneal dialysis patients on polymorphonuclear leukocyte functions. J Am Soc Nephrol (1995) 6:1592–9. 10.1681/ASN.V661592 8749685

[9] Cohen G Horl WH. Free immunoglobulin light chains as a risk factor in renal and extrarenal complications. Semin Dial (2009) 22:369–72. 10.1111/j.1525-139X.2009.00582.x 19708983

[10] Cohen G Rudnicki M Horl WH. Uremic toxins modulate the spontaneous apoptotic cell death and essential functions of neutrophils. Kidney Int Suppl (2001) 78:S48–52. 10.1046/j.1523-1755.2001.07818.x 11168982

[11] Dispenzieri A Katzmann JA Kyle RA Larson DR Therneau TM Colby CL. Use of nonclonal serum immunoglobulin free light chains to predict overall survival in the general population. Mayo Clin Proc (2012) 87:517–23. 10.1016/j.mayocp.2012.03.009 PMC3538473 22677072

[12] Hutchison CA Harding S Hewins P Mead GP Townsend J Bradwell AR. Quantitative assessment of serum and urinary polyclonal free light chains in patients with chronic kidney disease. Clin J Am Soc Nephrol (2008) 3:1684–90. 10.2215/CJN.02290508 PMC2572283 18945993

[13] Fenton A Jesky MD Webster R Stringer SJ Yadav P Chapple I. Association between urinary free light chains and progression to end stage renal disease in chronic kidney disease. PloS One (2018) 13:e197043. 10.1371/journal.pone.0197043 PMC5942781 29742142

[14] Holdsworth SR Summers SA. Role of mast cells in progressive renal diseases. J Am Soc Nephrol (2008) 19:2254–61. 10.1681/ASN.2008010015 18776124

[15] Haynes R Hutchison CA Emberson J Dasgupta T Wheeler DC Townend JN. Serum free light chains and the risk of ESRD and death in CKD. Clin J Am Soc Nephrol (2011) 6:2829–37. 10.2215/CJN.03350411 PMC3255360 22034503

[16] Knauf F Brewer JR Flavell RA. Immunity, microbiota and kidney disease. Nat Rev Nephrol (2019) 15:263–74. 10.1038/s41581-019-0118-7 30796361

[17] Mattiello F Verbist B Faust K Raes J Shannon WD Bijnens L. A web application for sample size and power calculation in case-control microbiome studies. Bioinformatics (2016) 32:2038–40. 10.1093/bioinformatics/btw099 27153704

[18] Mueller S Saunier K Hanisch C Norin E Alm L Midtvedt T. Differences in fecal microbiota in different European study populations in relation to age, gender, and country: a cross-sectional study. Appl Environ Microbiol (2006) 72:1027–33. 10.1128/AEM.72.2.1027-1033.2006 PMC1392899 16461645

[19] Wouters OJ O’Donoghue DJ Ritchie J Kanavos PG Narva AS. Early chronic kidney disease: diagnosis, management and models of care. Nat Rev Nephrol (2015) 11:491–502. 10.1038/nrneph.2015.85 26055354 PMC4531835

[20] Chapter 1: Definition and classification of CKD. Kidney Int Suppl 2011 (2013) 3:19–62. 25018975 10.1038/kisup.2012.64 PMC4089693

[21] Perez-Gomez MV Bartsch LA Castillo-Rodriguez E Fernandez-Prado R Fernandez-Fernandez B Martin-Cleary C. Clarifying the concept of chronic kidney disease for non-nephrologists. Clin Kidney J (2019) 12:258–61. 10.1093/ckj/sfz007 PMC6452188 30976406

[22] Xu B Tang Y Zhou J Zhang P Li H. Disease spectrum of abnormal serum free light chain ratio and its diagnostic significance. Oncotarget (2017) 8:82268–79. 10.18632/oncotarget.19391 PMC5669888 29137262

[23] Bolyen E Rideout JR Dillon MR Bokulich NA Abnet CC Al-Ghalith GA. Reproducible, interactive, scalable and extensible microbiome data science using QIIME 2. Nat Biotechnol (2019) 37:852–7. 10.1038/s41587-019-0209-9 PMC7015180 31341288

[24] Quast C Pruesse E Yilmaz P Gerken J Schweer J Yarza P. The SILVA ribosomal RNA gene database project improved data processing and web-based tools. Nucleic Acids Res (2013) 41(1):590–95. 10.1093/nar/gks1219 PMC3531112 23193283

[25] Rindlisbacher B Schild C Egger F Bacher V Pabst T Leichtle A. Serum Free Light Chain Assay: Shift Toward a Higher κ/λ Ratio. J Appl Lab Med (2020) 5:114–25. 10.1093/jalm.2019.029330 32445339

[26] Collins AJ Vassalotti JA Wang C Li S Gilbertson DT Liu J. Who should be targeted for CKD screening*?* Impact of diabetes, hypertension, and cardiovascular disease. Am J Kidney Dis (2009) 53:S71–7. 10.1053/j.ajkd.2008.07.057 19231764

[27] Garg AX Papaioannou A Ferko N Campbell G Clarke JA Ray JG. Estimating the prevalence of renal insufficiency in seniors requiring long-term care. Kidney Int (2004) 65:649–53. 10.1111/j.1523-1755.2004.00412.x 14717937

[28] He H Xie Y. Effect of Different Hemodialysis Methods on Microbiota in Uremic Patients. BioMed Res Int (2020) 2020:6739762. 10.1155/2020/6739762 32685517 PMC7321504

[29] Li F Wang M Wang J Li R Zhang Y. Alterations to the Gut Microbiota and Their Correlation With Inflammatory Factors in Chronic Kidney Disease. Front Cell Infect Microbiol (2019) 9:206. 10.3389/fcimb.2019.00206 31245306 PMC6581668

[30] Desjardins L Liabeuf S Lenglet A Lemke HD Vanholder R Choukroun G. Association between free light chain levels, and disease progression and mortality in chronic kidney disease. Toxins (Basel) (2013) 5:2058–73. 10.3390/toxins5112058 PMC3847714 24217396

[31] Sprangers B Claes K Evenepoel P Kuypers D Poesen K Delforge M. Comparison of 2 Serum-Free Light-Chain Assays in CKD Patients. Kidney Int Rep (2020) 5:627–31. 10.1016/j.ekir.2020.01.019 PMC7210599 32405584

[32] Ranganathan N Friedman EA Tam P Rao V Ranganathan P Dheer R. Probiotic dietary supplementation in patients with stage 3 and 4 chronic kidney disease: a 6-month pilot scale trial in Canada. Curr Med Res Opin (2009) 25:1919–30. 10.1185/03007990903069249 19558344

[33] Ranganathan N Ranganathan P Friedman EA Joseph A Delano B Goldfarb DS. Pilot study of probiotic dietary supplementation for promoting healthy kidney function in patients with chronic kidney disease. Adv Ther (2010) 27:634–47. 10.1007/s12325-010-0059-9 20721651

[34] Borges NA Carmo FL Stockler-Pinto MB de Brito JS Dolenga CJ Ferreira DC. Probiotic Supplementation in Chronic Kidney Disease: A Double-blind, Randomized, Placebo-controlled Trial. J Ren Nutr (2018) 28:28–36. 10.1053/j.jrn.2017.06.010 28888762

[35] Iwashita Y Ohya M Yashiro M Sonou T Kawakami K Nakashima Y. Dietary Changes Involving Bifidobacterium longum and Other Nutrients Delays Chronic Kidney Disease Progression. Am J Nephrol (2018) 47:325–32. 10.1159/000488947 29779028

[36] Wang X Yang S Li S Zhao L Hao Y Qin J. Aberrant gut microbiota alters host metabolome and impacts renal failure in humans and rodents. Gut (2020) 69(12):2131–42. 10.1136/gutjnl-2019-319766 PMC7677483 32241904

[37] Erdem BK Davran F Yilmaz VT Cetinkaya R Akbas H. The association of serum-free light-chain levels with markers of renal function. Ren Fail (2015) 37:1057–60. 10.3109/0886022X.2015.1052980 26056734

[38] Jiang S Xie S Lv D Wang P He H Zhang T. Alteration of the gut microbiota in Chinese population with chronic kidney disease. Sci Rep (2017) 7:2870. 10.1038/s41598-017-02989-2 28588309 PMC5460291

[39] Mariathasan S Weiss D Newton K McBride JO Rourke K Gm R. Cryopyrin activates the inflammasome in response to toxins and ATP. Nature (2006) 440:228–32. 10.1038/nature04515 16407890

[40] Lee LY Hook M Haviland D Wetsel RA Yonter EO Syribeys P. Inhibition of complement activation by a secreted Staphylococcus aureus protein. J Infect Dis (2004) 190:571–9. 10.1086/422259 15243934

[41] George B You D Joy MS Aleksunes LM. Xenobiotic transporters and kidney injury. Adv Drug Deliv Rev (2017) 116:73–91. 10.1016/j.addr.2017.01.005 28111348 PMC5519456

[42] Erridge C Bennett-Guerrero E Poxton IR. Structure and function of lipopolysaccharides. Microbes Infect (2002) 4:837–51. 10.1016/S1286-4579(02)01604-0 12270731

[43] Albillos A De-la-Hera A Alvarez-Mon M. Serum lipopolysaccharide-binding protein prediction of severe bacterial infection in cirrhotic patients with ascites. Lancet (2004) 363:1608–10. 10.1016/S0140-6736(04)16206-5 15145636

[44] Gaini S Koldkjaer OG Pedersen C Pedersen SS. Procalcitonin, lipopolysaccharide-binding protein, interleukin-6 and C-reactive protein in community-acquired infections and sepsis: a prospective study. Crit Care (2006) 10:R53. 10.1186/cc4866 16569262 PMC1550885

[45] Kitchens RL Thompson PA Munford RS O’Keefe GE. Acute inflammation and infection maintain circulating phospholipid levels and enhance lipopolysaccharide binding to plasma lipoproteins. J Lipid Res (2003) 44:2339–48. 10.1194/jlr.M300228-JLR200 12923224

[46] Campbell JP Eijsvogels TM Wang Y Hopman MT Jacobs JF. Assessment of serum free light chain levels in healthy adults immediately after marathon running. Clin Chem Lab Med (2016) 54:459–65. 10.1515/cclm-2015-0431 26351935

[47] McCullough PA Chinnaiyan KM Gallagher MJ Colar JM Geddes T Gold JM. Changes in renal markers and acute kidney injury after marathon running. Nephrology (Carlton) (2011) 16:194–9. 10.1111/j.1440-1797.2010.01354.x 21272132

[48] Liss MA White JR Goros M Gelfond J Leach R Johnson-Pais T. Metabolic Biosynthesis Pathways Identified from Fecal Microbiome Associated with Prostate Cancer. Eur Urol (2018) 74:575–82. 10.1016/j.eururo.2018.06.033 PMC6716160 30007819

[49] Xu Z Xie Z Sun J Huang S Chen Y Li C. Gut Microbiome Reveals Specific Dysbiosis in Primary Osteoporosis. Front Cell Infect Microbiol (2020) 10:160. 10.3389/fcimb.2020.00160 32373553 PMC7186314

[50] Petersen C Wankhade UD Bharat D Wong K Mueller JE Chintapalli SV. Dietary supplementation with strawberry induces marked changes in the composition and functional potential of the gut microbiome in diabetic mice. J Nutr Biochem (2019) 66:63–9. 10.1016/j.jnutbio.2019.01.004 PMC6490960 30771735

[51] Kiely CJ Pavli P O’Brien CL. The role of inflammation in temporal shifts in the inflammatory bowel disease mucosal microbiome. Gut Microbes (2018) 9:477–85. 10.1080/19490976.2018.1448742 PMC6287691 29543557

[52] Birn H Spiegelstein O Christensen EI Finnell RH. Renal tubular reabsorption of folate mediated by folate binding protein 1. J Am Soc Nephrol (2005) 16:608–15. 10.1681/ASN.2004080711 15703271

