# Supplementary figures and images for: Alteration of the Gut Microbiome in Chronic Kidney Disease Patients and Its Association With Serum Free Immunoglobulin Light Chains
Source: Front Immunol. 2021 Apr 1;12:609700. doi: 10.3389/fimmu.2021.609700 (PMC8047322; doi:10.3389/fimmu.2021.609700)

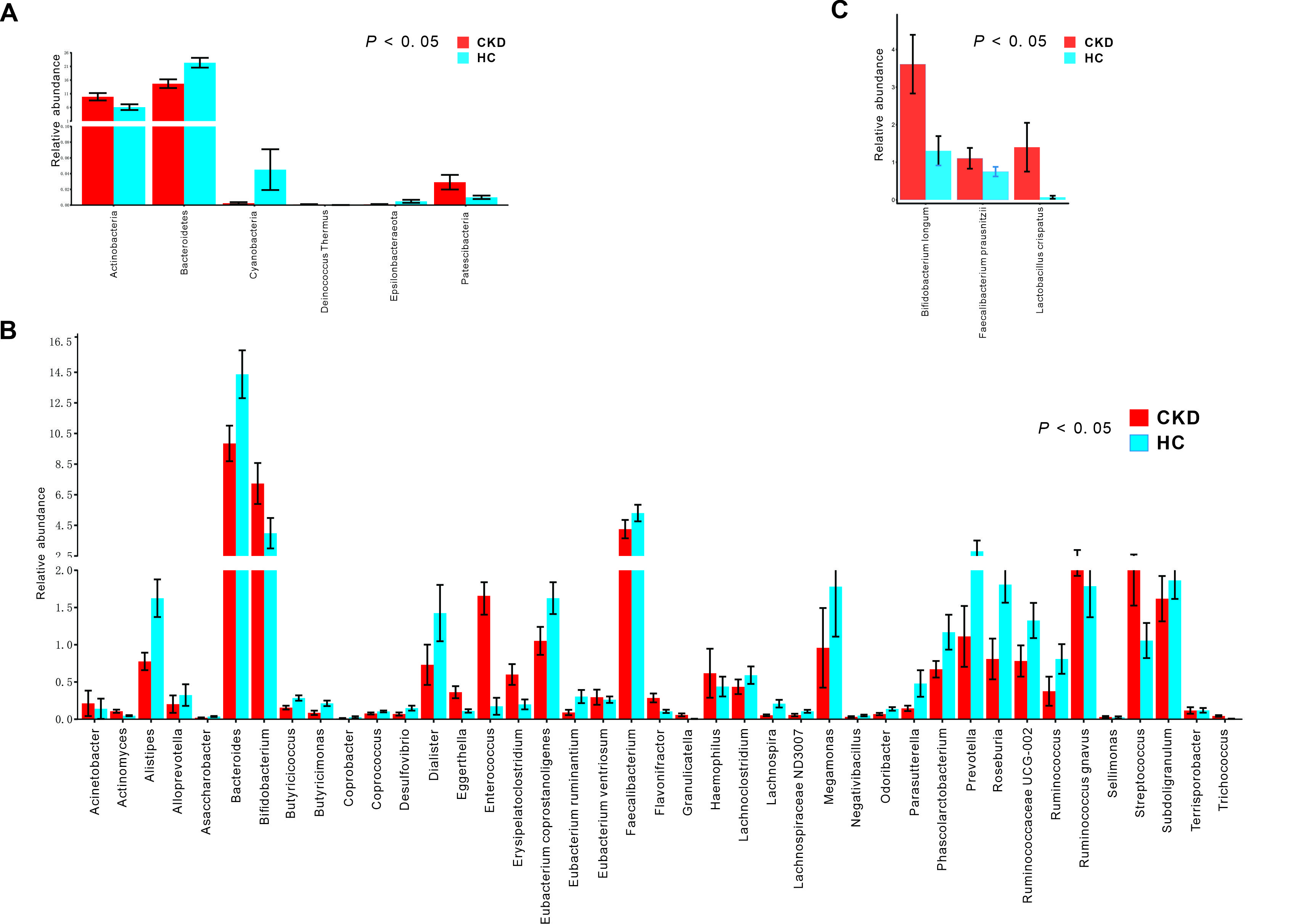

Supplement: Supplementary Figure 1 — Principal coordinates analysis (PCoA) revealed clustering of bacterial taxa in the six stages of CKD based on Bray–Curtis distance, with each point corresponding to a subject and colored according to the sample type. Permutational multivariate analysis of variance showed that the separation of bacterial communities in the six stages was not significant (P > 0.05). [file Image_1.jpeg]

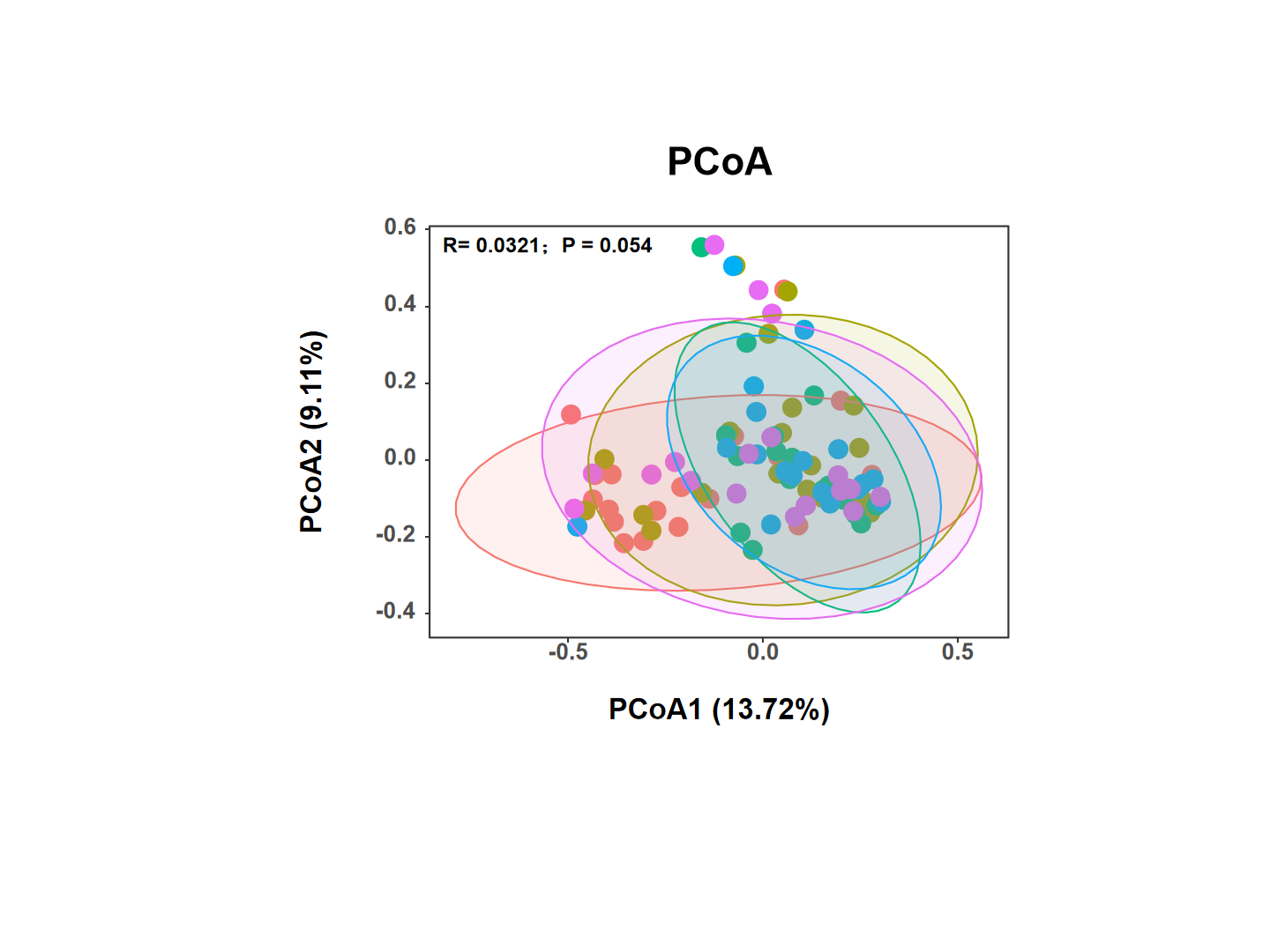

Supplement: Supplementary Figure 2 — Bacterial taxon abundance showing a significant difference between the CKD and HC groups without adjusting confounders (P < 0.05). The Wilcoxon rank-sum test was used to compare differences in abundance between the two groups. [file Image_2.tif]
