# Supplementary material for: Alteration of the Gut Microbiome in Chronic Kidney Disease Patients and Its Association With Serum Free Immunoglobulin Light Chains
Source: Front Immunol. 2021 Apr 1;12:609700. doi: 10.3389/fimmu.2021.609700 (PMC8047322; doi:10.3389/fimmu.2021.609700)
Supplement: Supplementary file 3 [file DataSheet_1.pdf]

SUPPLEMENTARY MATERIAL

Table S1 Comparison of metabolic pathways between the CKD and HC groups

Table S2 Characteristics of patients in CKD stages

Figure S1 Principal coordinates analysis (PCoA) revealed clustering of bacterial taxa in the six stages of CKD based on Bray–Curtis distance, with each point corresponding to a subject and colored according to the sample type. Permutational multivariate analysis of variance showed that the separation of bacterial communities in the six stages was not significant ($P > 0.05$).

Figure S2 Bacterial taxon abundance showing a significant difference between the CKD and HC groups without adjusting confounders ($P < 0.05$). The Wilcoxon rank-sum test was used to compare differences in abundance between the two groups.
