# Supplementary material for: Alteration of the Gut Microbiome in Chronic Kidney Disease Patients and Its Association With Serum Free Immunoglobulin Light Chains
Source: Front Immunol. 2021 Apr 1;12:609700. doi: 10.3389/fimmu.2021.609700 (PMC8047322; doi:10.3389/fimmu.2021.609700)
Supplement: Supplementary file 4 [file Table_1.pdf]

Table S1 Comparison of metabolic pathways between the CKD and HC groups

KEGG pathway	CKD	HC	*P*-value
Amino acid metabolism	0.23 ± 0.05	0.22 ± 0.05	0.037
Antigen processing and presentation	0.03 ± 0.01	0.04 ± 0.01	0.009
Ascorbate and aldarate metabolism	0.17 ± 0.08	0.15 ± 0.08	0.021
Bacterial chemotaxis	0.35 ± 0.12	0.43 ± 0.14	0.000
Bacterial motility proteins	0.69 ± 0.33	0.84 ± 0.30	0.000
Bacterial toxins	0.12 ± 0.04	0.11 ± 0.03	0.000
Biosynthesis of ansamycins	0.12 ± 0.02	0.12 ± 0.02	0.017
Biosynthesis of vancomycin group antibiotics	0.06 ± 0.01	0.06 ± 0.01	0.016
Biotin metabolism	0.15 ± 0.03	0.16 ± 0.03	0.019
Carbohydrate digestion and absorption	0.01 ± 0.01	0.02 ± 0.01	0.035
Carbon fixation pathways in prokaryotes	0.91 ± 0.07	0.94 ± 0.06	0.014
Cellular antigens	0.03 ± 0.02	0.03 ± 0.02	0.045
Chaperones and folding catalysts	0.94 ± 0.06	0.97 ± 0.06	0.001
Chloroalkane and chloroalkene degradation	0.24 ± 0.04	0.19 ± 0.03	< 0.001
Chlorocyclohexane and chlorobenzene degradation	0.02 ± 0.01	0.01 ± 0.01	0.022
Chromosome	1.48 ± 0.11	1.52 ± 0.12	0.002
Citrate cycle (TCA cycle)	0.54 ± 0.07	0.57 ± 0.07	0.014
Cyanoamino acid metabolism	0.30 ± 0.05	0.32 ± 0.07	0.011
Dioxin degradation	0.08 ± 0.03	0.07 ± 0.03	0.030
Energy metabolism	0.81 ± 0.13	0.85 ± 0.08	0.022
Fatty acid metabolism	0.27 ± 0.07	0.25 ± 0.07	0.006
Flagellar assembly	0.27 ± 0.16	0.34 ± 0.15	0.001
Flavone and flavonol biosynthesis	0.00 ± 0.01	0.01 ± 0.01	0.001
Germination	0.03 ± 0.02	0.04 ± 0.02	0.008
Glycerolipid metabolism	0.42 ± 0.05	0.42 ± 0.04	0.035
Glycolysis / Gluconeogenesis	1.22 ± 0.11	1.17 ± 0.08	0.001
Lipid biosynthesis proteins	0.54 ± 0.04	0.55 ± 0.04	0.021
Lipopolysaccharide biosynthesis	0.16 ± 0.12	0.19 ± 0.10	0.005
Lipopolysaccharide biosynthesis proteins	0.29 ± 0.16	0.32 ± 0.13	0.028
Membrane and intracellular structural molecules	0.48 ± 0.18	0.52 ± 0.15	0.027
Naphthalene degradation	0.15 ± 0.03	0.14 ± 0.02	0.007
Nitrogen metabolism	0.71 ± 0.06	0.72 ± 0.05	0.017
NOD-like receptor signaling pathway	0.03 ± 0.01	0.04 ± 0.01	0.025
One carbon pool by folate	0.57 ± 0.07	0.58 ± 0.08	0.009
Others	1.02 ± 0.1	0.99 ± 0.06	0.013
Pathways in cancer	0.04 ± 0.01	0.04 ± 0.01	0.002
Pentose phosphate pathway	0.92 ± 0.06	0.90 ± 0.05	0.007
Phosphotransferase system	0.87 ± 0.32	0.70 ± 0.30	0.000
Plant-pathogen interaction	0.14 ± 0.02	0.15 ± 0.03	0.001
Pores ion channels	0.32 ± 0.16	0.34 ± 0.13	0.024
Progesterone-mediated oocyte maturation	0.03 ± 0.01	0.04 ± 0.01	0.009
Propanoate metabolism	0.55 ± 0.05	0.53 ± 0.05	0.015

Prostate cancer	0.04 ± 0.01	0.04 ± 0.01	0.018
Protein processing in endoplasmic reticulum	0.05 ± 0.01	0.05 ± 0.02	0.015
Pyruvate metabolism	1.09 ± 0.07	1.07 ± 0.06	0.019
Replication, recombination and repair proteins	0.81 ± 0.1	0.77 ± 0.07	0.040
Restriction enzyme	0.16 ± 0.04	0.17 ± 0.03	0.012
Riboflavin metabolism	0.20 ± 0.03	0.22 ± 0.03	0.005
RNA polymerase	0.16 ± 0.03	0.15 ± 0.03	0.024
Signal transduction mechanisms	0.52 ± 0.04	0.50 ± 0.04	0.002
Staphylococcus aureus infection	0.02 ± 0.03	0.01 ± 0.02	0.006
Synthesis and degradation of ketone bodies	0.04 ± 0.01	0.03 ± 0.01	0.013
Tetracycline biosynthesis	0.15 ± 0.04	0.14 ± 0.02	0.008
Transcription factors	2.01 ± 0.27	1.93 ± 0.29	0.020
Transporters	7.62 ± 0.86	7.24 ± 0.86	0.002
Tropane, piperidine and pyridine alkaloid biosynthesis	0.11 ± 0.01	0.12 ± 0.01	0.008
Two-component system	1.43 ± 0.38	1.50 ± 0.31	0.028
Tyrosine metabolism	0.36 ± 0.05	0.35 ± 0.04	0.020
Vitamin B6 metabolism	0.19 ± 0.02	0.20 ± 0.02	0.026
Xylene degradation	0.07 ± 0.02	0.06 ± 0.02	0.048
Zeatin biosynthesis	0.05 ± 0.01	0.05 ± 0.01	0.033

The relative abundance of metabolic pathways ($\log_2$ transformed) was compared between the CKD and HC groups using the Wilcoxon rank test. Adjusted $P < 0.05$ was a cut-off as a significant difference.
