# Supplementary material for: Alteration of the Gut Microbiome in Chronic Kidney Disease Patients and Its Association With Serum Free Immunoglobulin Light Chains
Source: Front Immunol. 2021 Apr 1;12:609700. doi: 10.3389/fimmu.2021.609700 (PMC8047322; doi:10.3389/fimmu.2021.609700)
Supplement: Supplementary file 5 [file Table_2.doc]

**Table S2 Characteristics of patients in CKD stages**

| Parameters | | Stage 1  (n = 24) | Stage 2  (n = 20) | Stage 3  (n = 20) | Stage 4  (n = 18) | Stage 5  (n = 18) | P value |
| --- | --- | --- | --- | --- | --- | --- | --- |
| Gender | |  |  |  |  |  | 0.303 |
|  | Male | 8 | 13 | 11 | 10 | 9 |  |
|  | Female | 16 | 7 | 9 | 8 | 9 |  |
| Age | | 47.67 ± 15.42 | 56.00 ± 15.80 | 53.40 ± 17.79 | 64.67 ± 17.58 | 64.89 ± 14.21 | 0.003 |
| eGFR (mL / min / 1.73m^2^) | | 111.82 ± 20.84 | 73.25 ± 9.40 | 46.93 ± 8.23 | 24.19 ± 4.31 | 9.60 ± 2.63 | < 0.001 |
| Body mass index (kg / m^2^) | | 24.49 ± 3.86 | 24.08 ± 2.71 | 25.27 ± 4.27 | 26.39 ± 3.30 | 25.48 ± 3.40 | 0.301 |
| Serum FLC κ (g / L) | | 8.06 ± 2.44 | 8.56 ± 4.88 | 9.39 ± 2.22 | 9.30 ± 1.95 | 9.93 ± 2.23 | 0.278 |
| Serum FLC λ (g / L) | | 4.61 ± 1.43 | 4.67 ± 2.42 | 4.91 ± 1.41 | 4.71 ± 0.88 | 5.16 ± 1.08 | 0.802 |
| Serum κ / λ ratio | | 1.79 ± 0.37 | 1.89 ± 0.37 | 2.45 ± 2.78 | 1.99 ± 0.29 | 1.97 ± 0.47 | 0.525 |
| Serum urea (mmol / L） | | 12.48 ± 9.94 | 13.34 ± 14.38 | 10.49 ± 9.06 | 14.57 ± 12.99 | 15.16 ± 13.89 | 0.775 |
| Serum creatinine (umol / L) | | 232.09 ± 182.83 | 224.03 ± 215.66 | 164.05 ± 148.19 | 146.05 ± 119.41 | 149.44 ± 210.06 | 0.418 |
| Serum uric acid (umol / L) | | 362.73 ± 95.09 | 394.40 ± 99.58 | 436.02 ± 181.52 | 489.96 ± 169.15 | 445.22 ± 121.63 | 0.038 |
| Serum endotoxin (EU / mL) | | 0.10 ± 0.03 | 0.10 ± 0.05 | 0.10 ± 0.04 | 0.12 ± 0.03 | 0.09 ± 0.02 | 0.536 |

Pearson’s Chi-square/Fisher’s exact test was used to compare dichotomous variables, and one way ANOVA test was used to compare continuous variables.
